# Lipid Aberrations in Lichen Planus

**DOI:** 10.3390/metabo12111008

**Published:** 2022-10-22

**Authors:** Julia Nowowiejska, Anna Baran, Iwona Flisiak

**Affiliations:** Department of Dermatology and Venereology, Medical University of Bialystok, 15-540 Bialystok, Poland

**Keywords:** lichen planus, metabolic syndrome, lipids, lipid aberrations, atherosclerosis, statins

## Abstract

Lichen planus (LP) is a dermatosis without a fully understood etiopathogenesis, the frequency of which is estimated to be less than 1% among the population. LP may involve the glabrous skin, mucosal membranes, scalp, nails and genital area. Nowadays, there are reports of its association with lipid homeostasis aberrations. In this review, we present the contemporary view of this matter. Dyslipidemia, especially hypertriglyceridemia, seems to be an actual problem in this group of patients, and along with abnormal arterial vessel parameters, indicates an increased risk of atherosclerosis in these subjects. Dermatologists should be attentive to this relationship and aware that the patients may develop different metabolic complications. More studies are required to establish clear guidelines on the management of lipid aberrations in lichen planus.

## 1. Introduction

Lichen planus (LP) is a dermatosis without a fully understood etiopathogenesis, the frequency of which is estimated to be about 1% among the population [1,2,3]. Women and men seem to be equally affected; however, the disease is rare in children [4]. The exact cause of LP is not fully understood, but different factors have been taken into account [2]. The genetic background has been postulated to be involved, along with autoimmune mechanisms, and several triggers have been identified, such as drug intake, infections and psychological stress [5].

**Figure 1:**
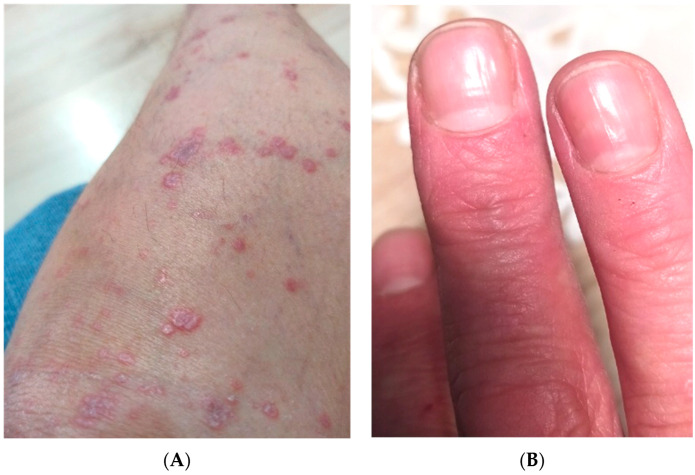
(**A**) Numerous polygonal pinkish-red papules with shiny Wickham striae located on the shin. (**B**) Longitudinal fissuring on the fingernail plates.

LP may involve the glabrous skin, mucosal membranes, scalp, nails and genital area [6,7,8]. Skin lesions are the most frequently observed manifestation and there are numerous variants of cutaneous LP [8]. The classic variant is characterized by the presence of red-violaceous polygonal papules with Wickham striae on their surface, which may be located anywhere on the body, but the most prevalent localizations are flexor surfaces of the limbs [4,8] (Figure 1A). Skin lesions are often accompanied by pruritus [4]. In approximately half of LP cases, the oral mucosa is involved [9]. There are many variants, but this usually manifests as white lace-like streaks [4]. Nails are involved in about 10% of cases and show a variety of abnormalities, including longitudinal fissuring, onycholysis, onychorrhexis and pterygium (Figure 1B) [4,9]. The scalp involvement is called lichen planopilaris (LPP) and manifests as perifollicular papules and scaling, finally leading to scarring alopecia [4,9].

LP may be associated with decreased quality of life, especially in cases of genital or eruptive lesions [10]. Recently, a new severity assessment tool has been introduced for LP [11].

The diagnosis of LP is mainly clinical, but well supported by dermoscopy and histopathological examination of the skin sample [4,9].

There are many treatment options for LP, usually dependent on the severity of the lesions and their localization. Commonly, the therapy involves topical or systemic glucocorticoids, phototherapy and oral retinoids [12].

Recent literature data highlighted that patients with LP may be affected above average by various comorbidities inter alia diabetes mellitus (DM), obesity, arterial hypertension and other cardiometabolic disorders (CMDs) [13,14,15].

This review summarizes the wide-ranging lipid aberrations reported in subjects with LP to provide an overview for scientists, identifies current research gaps and future research directions and draws physicians’ attention to metabolic disorders in such patients.

## 2. Materials and Methods

We performed a discursive review of the aberrations of lipid expression and metabolism in patients with lichen planus. We searched the PubMed database using the following MeSH: ‘lichen planus’ and ‘lipids’ or ‘fatty acids’ or ‘metabolic syndrome’ or ‘obesity’ or ‘lipid profile’ or ‘cholesterol’ or ‘triglycerides’ or ‘lipoproteins’ or ‘atherosclerosis’ or ‘non-alcoholic fatty liver disease’ or ‘fatty-acid binding proteins’ or ‘peroxisome proliferator-activated receptors’ or ‘adipokines’, without date limitations. Papers in the following languages were considered: English, Polish, German and French. The whole paper was read if the abstract indicated the relevance of the content.

## 3. Prevalence of Metabolic Disorders in Patients with Lichen Planus

The idea of the association of LP with metabolic disorders followed by cardiovascular complications derives from the inflammatory nature of this condition, mediated by T-lymphocytes [7]. It has been proposed that chronic inflammation leads to lipid homeostasis aberrations and resulting implications [7]. Moreover, LP is similar to psoriasis in several aspects, and the latter has been closely associated with metabolic disorders [16].

### 3.1. Metabolic Syndrome

Metabolic syndrome (MS) is defined, according to the American Heart Association, as the presence of three out of five criteria: increased waist circumference of 35 inches in females and 40 inches in males; triglyceride serum concentration > 150 mg/dL or treatment for hyperglyceridemia; HDL-C (high-density lipoprotein) serum concentration < 40 mg/dL in males and <50 mg/dL in females or treatment for this disorder; fasting blood glucose of 100 mg/dL or treatment for type 2 diabetes mellitus; systolic blood pressure of 130 mmHg, diastolic blood pressure of 85 mm or treatment for hypertension [17]. It has been associated with many different dermatoses, especially psoriasis, but also vitiligo [13], hidradenitis suppurativa [5] and alopecia [18]. Nowadays, there are also reports of its association with LP. 

There are several studies investigating the exact prevalence of MS in patients with LP. The reported frequency of MS in such patients is so far quite broad, ranging between 6 and 77% [1]. We found four studies in which the frequency of MS in patients with LP was statistically significantly higher than in persons without this dermatosis [13,19,20,21]. We also found another three investigations, but with insignificantly higher reports for the prevalence of MS in such patients [1,18,22], and one study that reported the frequency of MS to be 35.7%, but with no control group [14].

A large study by Dreiher et al., which investigated different components of MS in 1477 patients with LP, revealed that there was only a statistically significant difference in the prevalence of dyslipidemia between the groups. There was no significant difference for obesity, DM or arterial hypertension [23]. Noteworthy, in another study, MS occurred less frequently in patients with Wickham’s striae [1]. A second interesting observation was that the family history of DM could be an independent predictive factor for the occurrence of MS in LP patients [1]. In one study, the duration of LP was insignificantly higher in patients with MS than in those without [14].

### 3.2. Atherosclerosis

There is also research regarding atherosclerosis in LP subjects. Three studies aimed to assess, inter alia, the carotid intima media thickness, which is an established marker of subclinical atherosclerosis, in patients with LP compared to controls [20,24,25]. They proved that it is indeed significantly thicker in patients, despite no clinical evidence of heart disease, than in persons without LP [20,24,25]. In one of these studies, the impairment of flow-mediated dilatation of the brachial artery, which is also considered an early predictor of atherosclerosis, was studied and found to be significantly reduced in patients with LP than in controls [25]. Moreover, in further research, the white blood cell count, as an inflammatory indicator, was positively correlated with the carotid intima media thickness in patients, which may point to the role of systemic inflammation in metabolic complications for those with LP [24].

An interesting study was conducted by Ertem et al. It aimed at establishing the thickness of the epicardial fat tissue, which is the adipose tissue between the myocardial epicardium and visceral epicardium of the heart. The idea originated from the observation that epicardial fat tissue is a source of proatherogenic and proinflammatory hormones and cytokines, hence its association with metabolic disorders, and the fact that it is significantly increased in another dermatosis, psoriasis [2]. Indeed, they proved that patients with LP also have this abnormality, which makes them more prone to atherosclerosis [2].

Noteworthy, three studies reported significantly increased homocysteinemia in subjects with LP, which is another risk factor for atherosclerosis [19,20,26]. One team of scientists even suggested folic acid supplementation as counteracting homocysteinemia [19].

### 3.3. Non-Alcoholic Fatty Liver Disease

We have not found any report about non-alcoholic fatty liver disease (NAFLD) in patients with LP; therefore, this subject may require more attention in the future.

## 4. Aberrations of Blood Lipids in Patients with Lichen Planus

### 4.1. Cholesterol

In 2016, Lai et al. performed a big meta-analysis in order to investigate whether patients with LP are more prone to dyslipidemia. The analysis contained some heterogenicity because patients with skin involvement (different LP subtypes) and, in some cases, also with oral LP were enrolled. Nevertheless, patients with LP had higher, although insignificantly, total cholesterol concentrations. The same outcomes were obtained for low-density lipoprotein (LDL) concentrations, and as for HDL, its levels were insignificantly decreased [27].

As for other newer, independent investigations, nine studies assessed the total cholesterol concentration in patients with LP and controls. Seven of them revealed significantly increased total cholesterol concentrations in patients compared to controls [1,7,13,19,20,22,26,28], while only two studies showed insignificantly elevated levels [5,6].

Eleven studies compared the LDL concentrations between patients with LP and controls. Nine of them reported significantly elevated LDL in patients [1,7,13,19,20,22,24,26,28], while only two showed insignificant elevation [5,6].

Twelve studies evaluated HDL levels. In seven of them, the HDL concentration was significantly decreased in patients compared to controls [7,19,20,22,25,26,28], while in five studies, the HDL level was decreased but insignificantly [1,6,13,21,24].

Noteworthy, in one report, the total cholesterol level was negatively associated with the LP duration in patients [6].

Three studies also evaluated the LDL-C/HDL-C ratio, which has been suggested as a reliable predictor of cardiovascular complications, and found that it was significantly higher in patients with LP than in controls [7,18,19].

### 4.2. Triglycerides

The previously mentioned meta-analysis by Lai et al. revealed significantly increased triglyceride concentrations in patients with LP [27], similar to in more recent studies [1,13,20,21,22,24,26]. Two other studies revealed elevated triglycerides in patients with LP as well, but insignificantly [5,6].

### 4.3. Adipokines

One study showed that leptin serum concentrations are higher in patients with LP, although insignificantly, than in controls [6]. Moreover, the serum leptin level was positively correlated with the BMI [6]. Except for that study, we have not found any other mention of adipokines in LP, which suggests a need to fill the research gap.

### 4.4. Aberrations in Specific Lichen Planus Subtypes

#### 4.4.1. Oral Lichen Planus

The second most common manifestation of LP is oral mucosa involvement. There have been several studies on metabolic disorders and lipid aberrations in this particular subtype.

The prevalence of MS specifically in patients with oral LP was investigated in two studies. In the first study, it was significantly more frequent than in controls [29], and in the other, there was no difference [30]. A further finding in the first study was that in subjects with MS, the duration of LP was significantly longer [29].

We managed to find five studies on the total cholesterol concentration in patients with oral LP. In two of them, it was significantly higher than in controls without this dermatosis [30,31], and in the rest it was insignificantly higher [29,32,33]. There were no significant differences between particular oral LP clinical types, but the highest concentration of total cholesterol was found in subjects with the atrophic-erosive manifestation [32,33]. 

Four studies assessed LDL concentrations in patients with oral LP. In the majority of them, it was insignificantly higher than in the control group [29,30,31], and in just one study, it was insignificantly lower in patients than in controls [33].

HDL concentrations were investigated in five studies. Surprisingly, in the majority of them, they were insignificantly higher than in subjects without this dermatosis [29,30,31,32]; in only one investigation was the concentration significantly lower in patients than in controls [33].

Four studies analyzed triglyceride levels in patients with oral LP. In all of them, the level was higher than in controls [29,30,31,32,33], but only significantly in one case [30]. Interestingly, in two separate studies, the level of triglycerides significantly differed between the specific clinical subtypes of LP, seemingly the highest in the atrophic and erosive manifestation [32,33].

One study revealed that adiponectin concentrations were higher in subjects with oral LP [34].

#### 4.4.2. Lichen Planopilaris

In one paper investigating subjects solely with LPP, the total cholesterol, as well as LDL and HDL, appeared to be higher than in controls, but insignificantly [35]. In the same study, the triglyceride concentration was surprisingly found to be lower, although insignificantly, in patients than in controls [35]. 

#### 4.4.3. Nail Lichen Planus

We have not found any reports of lipid aberrations in nail LP exclusively, but we have to keep in mind that it is a less frequent manifestation and rarely occurs solely without other symptoms. It is also commonly underdiagnosed.

## 5. Aberrations of Lipids in Skin Lesions of Patients with Lichen Planus

We are not aware of any investigations performed on skin samples taken from lichenoid lesions. Such studies have been performed on psoriatic tissue and psoriatic scales [36,37], with some interesting results, so this could be a new research direction for LP.

**Figure 2:**
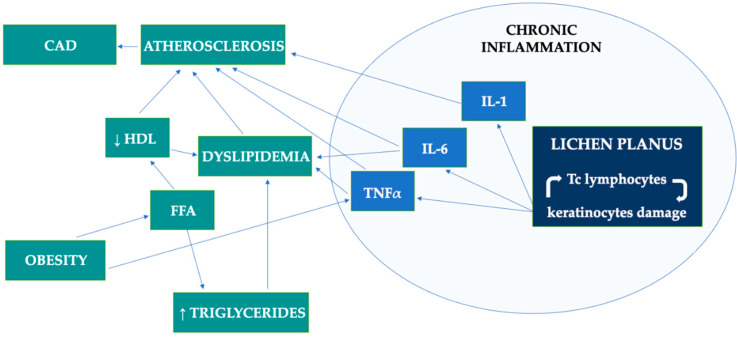
Potential pathomechanism of lipid aberrations and metabolic complications in patients with lichen planus. CAD, coronary artery disease; FFA, free fatty acids; IL, interleukin; Tc lymphocytes, cytotoxic T lymphocytes; TNF, tumor necrosis factor.

Figure 2 presents the potential pathomechanism of lipid aberrations and metabolic complications in patients with LP.

## 6. Influence of Lipid-Lowering Drugs on Lichen Planus

Statins, which are inhibitors of 3-hydroxy-3-methylo-glutarylo-coenzyme A, are no doubt the most commonly prescribed lipid-lowering agents [38]. Unfortunately, the available literature shows that they can trigger lichenoid eruptions in subjects treated for hyperlipidemia [39,40,41]. On the other hand, some scientists point to the antioxidant and anti-chemotactic properties of statins, along with their ability to inhibit leukocyte activation and proliferation, as well as cytokine release, which could be beneficial in the case of LP [42]. 

Fibrates are another group of lipid-lowering agents that bind to nuclear PPARα [43], but we were not able to find any reports on the influence of fibrates on LP.

Glitazones, which are PPARγ agonists, act due to the activation of these receptors, leading to the transcription of genes involved in the metabolism of fatty acids and decreasing their blood concentration [44]. There are several reports on glitazones in the context of LPP [45,46,47]. The idea to use these drugs in LPP originated from the suspicion that the inflammation-initiating factor in this alopecia may be an abnormal function of PPARγ leading to aberrations in the hair-sebaceous unit [45]. There are reports of the beneficial use of glitazones in the treatment of LPP [45,46,47]. 

## 7. Influence of Therapies Used in Lichen Planus on Lipid Homeostasis

Topical agents and phototherapy used in the treatment of LP obviously have no effect on lipids in this group of patients. However, two systemic drugs, namely acitretin and oral glucocorticoids, frequently used prednisone, have the ability to influence lipid homeostasis. Acitretin is well-known to affect lipid parameters. The side effects of its use are an increase in the triglyceride and cholesterol concentrations, and a decrease in HDL [48]. In cases of LPP, however, there are no clear guidelines on its treatment use. Isotretinoin is frequently used [49], which is another retinoid causing similar side effects to those described above for acitretin. As for systemic glucocorticoids, they are able to induce lipolysis and synthesis of VLDL, the release of free fatty acids and their accumulation in the liver [50]. However, the data regarding the serum lipid profile in subjects on glucocorticoid therapy are sometimes conflicting. Some papers report increased cholesterol and triglyceride levels, but there are also data to the contrary, even some showing that systemic glucocorticoids act positively, for instance, by potentially increasing HDL [51].

## 8. Discussion

A summary of the studies included in our review is presented in Table S1. Our literature search revealed that the majority of papers concerned cutaneous LP. The second most commonly investigated manifestation was oral mucosa LP. There is much less information about scalp and nail involvement. More studies are needed in patients solely with the oral or nail manifestation of LP to further our knowledge. Lipid aberrations in the group of patients with LPP have also been poorly studied so far and should be investigated in the future. 

Noteworthy, the countries of origin of subjects included in the available studies are fairly homogenous. Few studies have been performed on European or Southern American populations. The majority of studies have come from Asia or Africa. More studies on the European population would be appreciated. We did not observe fundamental differences between patients of various origins, so for now, it seems that ancestry does not really affect differences in lipid profiles among patients with LP.

The majority of investigations included at least 50 patients, which although at first seems a small sample size, should be perhaps considered sufficient considering LP is a rare dermatosis. Again, we did not observe that the sample size was related to particular results obtained in the studies. However, some inconsistencies between the different research studies, in our opinion, may have been caused by the different LP subtypes analyzed, together with factors such as their separation from others with the condition, their living area, diet and the technique used in the laboratory investigations. There is little information on the influence of another variable—the LP duration—on MS or dyslipidemia, but currently, it seems there is no relationship.

The current evidence indicates that dyslipidemia is a real problem in patients with LP, especially hypertriglyceridemia. Laboratory parameters such as the total cholesterol, triglycerides, LDL and HDL seem relatively easy and cheap investigations that could be performed by dermatologists or general practitioners on patients with LP in order to detect lipid homeostasis disturbances early on. Another useful tool could be the LDL-C/HDL-C ratio, which is considered a reliable predictor of cardiovascular complications and is quick and easy to calculate based on parameters obtained from laboratory tests. Dyslipidemia, along with abnormal arterial vessel parameters, indicates an increased risk of atherosclerosis in subjects with LP. However intriguing, measurement of the intima media thickness or epicardial adipose tissue parameters seems to have rather low chances for application in daily clinical practice considering the high cost of adequate equipment and the need for staff training.

As for the comparison between the two most frequent LP forms—oral and cutaneous—it seems that in both of them dyslipidemia is a threat, but it is more prominent in the cutaneous form. LPP seems to have less of a close relationship with dyslipidemia, but there are insufficient data on this as of yet.

When considering the drug influence of LP, the matter of the potential beneficial impact of statins on LP (despite the possibility of drug eruptions) will perhaps be verified in the future. It would be interesting to investigate the influence of statins regarding particular agents and different doses. Fibrates and glitazones could also be investigated as new therapeutic agents in this indication. Moreover, considering the above-mentioned lipid alterations due to retinoids or steroids, during the treatment for LP, we may encounter lipid aberrations aside from the ones already resulting from the dermatosis itself.

To note the limitations of our review, we came across certain obstacles. The main difficulty was in interpreting the available research related to the different manifestations of lichen planus that were taken into consideration. Some papers aimed to analyze only LP with skin involvement, some of them only oral LP and others both at the same time. Moreover, some studies included patients with several different subtypes of cutaneous LP. There were also studies on patients from different countries but where their ethnicity was not specified, which may have influenced the results. Furthermore, in some research, a weakness was the small sample size.

## 9. Conclusions

Lichen planus has emerged as a dermatosis associated with metabolic syndrome. The current evidence indicates that dyslipidemia is a real problem in patients with this dermatosis, especially hypertriglyceridemia. Dermatologists should be attentive to this relationship and aware that the patients may develop different metabolic complications. Perhaps simple laboratory investigations of lipid parameters in such patients or measurements of their waist circumference in daily dermatological practice could benefit these patients, but more studies are required to establish guidelines regarding such approaches. Aberrations in subjects with nail involvement, lichen planopilaris and non-alcoholic fatty liver disease in lichen planus patients seem to be very poorly investigated in the field of lichen planus research so far; hence, they may become future research targets.

## Supplementary Materials

The following supporting information can be downloaded at: https://www.mdpi.com/article/10.3390/metabo12111008/s1, Table S1. Summary of studies included in our review.

## References

[1] Okpala I.C., Akinboro A.O., Ezejoifor I.O., Onunu A.N., Okwara B.U. (2019). Metabolic Syndrome and Dyslipidemia among Nigerians with Lichen Planus: A Cross-Sectional Study. Indian J. Dermatol..

[2] Ertem A.G., Erdogan M., Koseoglu C., Akoglu G., Ozdemir E., Koseoglu G., Sivri S., Keles T., Durmaz T., Aktas A. (2016). Epicardial fat tissue thickness is increased in patients with lichen planus and is linked to inflammation and dyslipidemia. Rev. Port. De Cardiol..

[3] González-Moles M.Á., Warnakulasuriya S., González-Ruiz I., González-Ruiz L., Ayén Á., Lenouvel D., Ruiz-Ávila I., Ramos-García P. (2021). Worldwide prevalence of oral lichen planus: A systematic review and meta-analysis. Oral Dis..

[4] Gorouhi F., Davari P., Fazel N. (2014). Cutaneous and mucosal lichen planus: A comprehensive review of clinical subtypes, risk factors, diagnosis, and prognosis. Sci. World J..

[5] Özkur E., Uğurer E., Altunay İ.K. (2020). Dyslipidemia in Lichen Planus: A Case-control Study. Med. Bull. Sisli Etfal Hosp..

[6] Aryanian Z., Shirzadian A., Hatami P., Dadras H. (2022). High Incidence of Metabolic Syndrome Components in Lichen Planus Patients: A Prospective Cross-Sectional Study. Int. J. Clin. Pract..

[7] Kar B.R., Panda M., Patro N. (2016). Metabolic Derangements in Lichen Planus—A Case Control Study. J. Clin. Diagn. Res..

[8] Tziotzios C., Lee J.Y.W., Brier T., Saito R., Hsu C.K., Bhargava K., Stefanato C.M., Fenton D.A., McGrath J.A. (2018). Lichen planus and lichenoid dermatoses: Clinical overview and molecular basis. J. Am. Acad. Dermatol..

[9] Arnold D.L., Krishnamurthy K. (2022). Lichen Planus. StatPearls.

[10] Fiocco Z., Kupf S., Patzak L., Kämmerer T., Pumnea T., French L.E., Reinholz M. (2021). Quality of Life and Psychopathology in Lichen Planus: A Neglected Disease Burden. Acta Derm.-Venereol..

[11] Kaur H., Nikam B.P., Jamale V.P., Kale M.S. (2020). Lichen Planus Severity Index: A new, valid scoring system to assess the severity of cutaneous lichen planus. Indian J. Dermatol. Venereol. Leprol..

[12] Husein-ElAhmed H., Gieler U., Steinhoff M. (2019). Lichen planus: A comprehensive evidence-based analysis of medical treatment. J. Eur. Acad. Dermatol. Venereol..

[13] Singla R., Ashwini P.K., Jayadev B. (2019). Lichen Planus and Metabolic Syndrome: Is There a Relation?. Indian Dermatol. Online J..

[14] Hashba H., Bifi J., Thyvalappil A., Sridharan R., Sreenivasan A., Mathew P. (2018). Prevalence of Metabolic Syndrome in Patients with Lichen Planus: A Cross-sectional Study from a Tertiary Care Center. Indian Dermatol. Online J..

[15] Hasan S., Ahmed S., Kiran R., Panigrahi R., Thachil J.M., Saeed S. (2019). Oral lichen planus and associated comorbidities: An approach to holistic health. J. Fam. Med. Prim. Care..

[16] Baran A., Nowowiejska J., Kaminski T.W., Krahel J.A., Flisiak I. (2021). Circulating MAdCAM-1 and ITGB7 in Patients with Plaque Psoriasis and Eruptive Lichen Planus—Preliminary Data. Biology.

[17] American Heart Association Metabolic Syndrome Criteria. https://www.ahajournals.org/doi/10.1161/circulationaha.105.169404.

[18] Arias-Santiago S., Buendía-Eisman A., Aneiros-Fernández J., Girón-Prieto M.S., Gutiérrez-Salmerón M.T., García-Mellado V., Cutando A., Naranjo-Sintes R. (2011). Lipid levels in patients with lichen planus: A case-control study. J. Eur. Acad. Dermatol. Venereol..

[19] Saleh N., Samir N., Megahed H., Farid E. (2013). Homocysteine and other cardiovascular risk factors in patients with lichen planus. J. Eur. Acad. Dermatol. Venereol..

[20] Nasiri S., Sadeghzadeh-Bazargan A., Robati R.M., Haghighatkhah H.R., Younespour S. (2019). Subclinical atherosclerosis and cardiovascular markers in patients with lichen planus: A case–control study. Indian J. Dermatol. Venereol. Leprol..

[21] Daye M., Temiz S.A., Isık B. (2021). The relationship between lichen planus and metabolic syndrome. J. Cosmet. Dermatol..

[22] Kumar S.A., Krishnam Raju P.V., Gopal K.V.T., Rao T.N. (2019). Comorbidities in Lichen Planus: A Case-control Study in Indian Patients. Indian Dermatol. Online J..

[23] Dreiher J., Shapiro J., Cohen A.D. (2009). Lichen planus and dyslipidaemia: A case-control study. Br. J. Dermatol..

[24] Koseoglu C., Erdogan M., Ertem A.G., Koseoglu G., Akoglu G., Aktas A., Ozdemir E., Kurmus O., Durmaz T., Keles T. (2016). Aortic Elastic Properties and Myocardial Performance Index Are Impaired in Patients with Lichen Planus. Med. Princ. Pract..

[25] Aksu F., Karadag A.S., Caliskan M., Uzuncakmak T.K., Keles N., Ozlu E., Yilmaz Y., Akdeniz N. (2016). Does Lichen Planus Cause Increased Carotid Intima-Media Thickness and Impaired Endothelial Function?. Can. J. Cardiol..

[26] Rashed L., Abdel Hay R., AlKaffas M., Ali S., Kadry D., Abdallah S. (2017). Studying the association between methylenetetrahydrofolate reductase (MTHFR) 677 gene polymorphism, cardiovascular risk and lichen planus. J. Oral Pathol. Med..

[27] Lai Y.C., Yew Y.W., Schwartz R.A. (2016). Lichen planus and dyslipidemia: A systematic review and meta-analysis of observational studies. Int. J. Dermatol..

[28] Ozbagcivan O., Akarsu S., Semiz F., Fetil E. (2020). Comparison of serum lipid parameters between patients with classic cutaneous lichen planus and oral lichen planus. Clin. Oral Investig..

[29] Baykal L., Arıca D.A., Yaylı S., Örem A., Bahadır S., Altun E., Yaman H. (2015). Prevalence of Metabolic Syndrome in Patients with Mucosal Lichen Planus: A Case-Control Study. Am. J. Clin. Dermatol..

[30] Krishnamoorthy B., Suma G.N., Mamatha N.S., Sowbhagya M.B., Garlapati K. (2014). Lipid profile and metabolic syndrome status in patients with oral lichen planus, oral lichenoid reaction and healthy individuals attending a dental college in northern India—A descriptive study. J. Clin. Diagn. Res..

[31] Mehdipour M., Taghavi Zenouz A., Davoodi F., Gholizadeh N., Damghani H., Helli S., Safarnavadeh M. (2015). Evaluation of the Relationship between Serum Lipid Profile and Oral Lichen Planus. J. Dent. Res. Dent. Clin. Dent. Prospect..

[32] Toader M.P., Taranu T., Constantin M.M., Olinici D., Mocanu M., Costan V.V., Toader S. (2021). High serum level of interleukin-6 is linked with dyslipidemia in oral lichen planus. Exp. Ther. Med..

[33] López-Jornet P., Camacho-Alonso F., Rodríguez-Martínes M.A. (2012). Alterations in Serum Lipid Profile Patterns in Oral Lichen Planus. Am. J. Clin. Dermatol..

[34] Lopez-Jornet P., Cayuela C.A., Tvarijonaviciute A., Parra-Perez F., Escribano D., Ceron J. (2016). Oral lichen planus: Salival biomarkers cortisol, immunoglobulin A., adiponectin. J. Oral Pathol. Med..

[35] Conic R.R.Z., Piliang M., Bergfeld W., Atanaskova-Mesinkovska N. (2018). Association of Lichen Planopilaris With Dyslipidemia. JAMA Dermatol..

[36] Tekin N.S., Tekin I.O., Barut F., Sipahi E.Y. (2007). Accumulation of oxidized low-density lipoprotein in psoriatic skin and changes of plasma lipid levels in psoriatic patients. Mediat. Inflamm..

[37] Motta S., Sesana S., Ghidoni R., Monti M. (1995). Content of the different lipid classes in psoriatic scale. Arch. Dermatol. Res..

[38] Almeida S.O., Budoff M. (2019). Effect of statins on atherosclerotic plaque. Trends Cardiovasc. Med..

[39] Noël B. (2007). Lupus erythematosus and other autoimmune diseases related to statin therapy: A systematic review. J. Eur. Acad. Dermatol. Venereol..

[40] Stoebner P.E., Michot C., Ligeron C., Durand L., Meynadier J., Meunier L. (2003). Lichen plan pemphigoïde induit par la simvastatine [Simvastatin-induced lichen planus pemphigoides]. Ann. Dermatol. Venereol..

[41] Forouzan P., Riahi R.R., Cohen P.R. (2020). Atorvastatin-induced Lichenoid Drug Eruption: A Case Report and Review of Statin-associated Cutaneous Adverse Events. Cureus.

[42] Namazi M.R. (2004). Statins: Novel additions to the dermatologic arsenal?. Exp. Dermatol..

[43] Okopień B., Buldak L., Bołdys A. (2017). Fibrates in the management of atherogenic dyslipidemia. Expert Rev. Cardiovasc. Ther..

[44] Han L., Shen W.J., Bittner S., Kraemer F.B., Azhar S. (2017). PPARs: Regulators of metabolism and as therapeutic targets in cardiovascular disease. Part II: PPAR-β/δ and PPAR-γ. Future Cardiol..

[45] Mirmirani P., Karnik P. (2009). Lichen planopilaris treated with a peroxisome proliferator-activated receptor gamma agonist. Arch. Dermatol..

[46] Mesinkovska N.A., Tellez A., Dawes D., Piliang M., Bergfeld W. (2015). The use of oral pioglitazone in the treatment of lichen planopilaris. J. Am. Acad. Dermatol..

[47] Peterson E.L., Gutierrez D., Brinster N.K., Lo Sicco K.I., Shapiro J. (2019). Response of Lichen Planopilaris to Pioglitazone Hydrochloride. J. Drugs Dermatol..

[48] Sarkar R., Chugh S., Garg V.K. (2013). Acitretin in dermatology. Indian J. Dermatol. Venereol. Leprol..

[49] Babahosseini H., Tavakolpour S., Mahmoudi H., Balighi K., Teimourpour A., Ghodsi S.Z., Abedini R., Ghandi N., Lajevardi V., Kiani A. (2019). Lichen planopilaris: Retrospective study on the characteristics and treatment of 291 patients. J. Dermatol. Treat..

[50] Oray M., Abu Samra K., Ebrahimiadib N., Meese H., Foster C.S. (2016). Long-term side effects of glucocorticoids. Expert Opin. Drug Saf..

[51] Choi H.K., Seeger J.D. (2005). Glucocorticoid use and serum lipid levels in US adults: The Third National Health and Nutrition Examination Survey. J. Am. Coll. Rheumatol..

