# Supplementary material for: Lipid Aberrations in Lichen Planus
Source: Metabolites. 2022 Oct 22;12(11):1008. doi: 10.3390/metabo12111008 (PMC9693399; doi:10.3390/metabo12111008)
Supplement: Supplementary file 1 [file metabolites-12-01008-s001.zip › metabolites-1969054-supplementary.pdf]

Table S1. The summary of studies included into our review.

Cited Reference	Sample Size	Origin	Involvement by LP
[1]	90 patients; 90 controls	Nigeria	cutaneous, mucosal, nail
[5]	49 patients; 99 controls	Turkey	cutaneous, mucosal
[6]	40 patients; 40 controls	Iran	cutaneous, mucosal
[7]	40 patients; 40 controls	India	cutaneous, mucosal
[13]	100 patients; 50 controls	India	cutaneous, mucosal
[14]	70 patients; no controls	India	cutaneous, mucosal
[18]	80 patients; 80 controls	Spain	cutaneous, mucosal, nail, scalp
[19]	40 patients; 40 controls	Egypt	cutaneous
[20]	43 patients; 43 controls	Iran	cutaneous, mucosal, scalp
[21]	98 patients, 99 controls	Turkey	cutaneous, mucosal, nail, scalp
[22]	75 patients; 75 controls	India	cutaneous, mucosal, scalp
[23]	1477 patients; 2856 controls	Israel	not specified
[20]	54 patients; 50 controls	Turkey	cutaneous, mucosal
[25]	30 patients; 30 patients	Turkey	cutaneous
[26]	110 patients; 120 controls	Egypt	cutaneous, mucosal
[27]	110 patients; 120 controls	Egypt	cutaneous, mucosal
[28]	90 patients; 30 controls	Turkey	cutaneous, mucosal
[29]	79 patients; 79 controls	Turkey	cutaneous, mucosal, nail
[30]	18 patients; 14 controls	India	mucosal LP or oral lichenoid reactions
[31]	44 patients; 44 controls	Iran	mucosal
[32]	18 patients; 18 controls	Romania	mucosal
[33]	200 patients; 200 controls	Spain	mucosal
[34]	33 patients; 32 controls	Spain	mucosal
[35]	187 patients; 56 controls	USA	scalp
